# From Food Systems to Gut Microbiota: Dietary Substrates, Microbial Exposure and One Health

**DOI:** 10.3390/microorganisms14071482

**Published:** 2026-07-07

**Authors:** Inês R. Barreto, Ana Eugénio, Mário Cristóvão, Francisco Rodrigues, Christophe Espírito Santo, Inês Brandão

**Affiliations:** 1 Centro de Apoio Tecnológico Agro Alimentar (CATAA), 6000-459 Castelo Branco, Portugal; ines.barreto@cataa.pt (I.R.B.); anaeugenio@cataa.pt (A.E.); mario.cristovao@cataa.pt (M.C.); cespiritosanto@cataa.pt (C.E.S.); 2 Centre for Functional Ecology (CFE), Associate Laboratory TERRA, Department of Life Sciences, University of Coimbra, 3000-456 Coimbra, Portugal; 3 Dr. Lopes Dias Higher School of Health, Polytechnic University of Castelo Branco, 6000-084 Castelo Branco, Portugal; franciscobrodrigues@ipcb.pt; 4 SPRINT—Sport Physical Activity and Health Research and Innovation Center, Polytechnic University of Castelo Branco, 6000-084 Castelo Branco, Portugal

**Keywords:** One Health, food systems, gut microbiome, microbial exposure, microbiome resilience, soil–food–gut continuum, microbial stewardship

## Abstract

Food systems are usually discussed in terms of nutrition, food safety, productivity, sustainability or emissions. Less attention is given to the microbial dimension of the farm-to-fork pathway and to the way food systems shape the dietary substrates, food matrices and microbial exposures that reach the gut. Soils, plants, foods, processing environments, animals and the human gut all host microbial communities that influence nutrient cycling, plant performance, food characteristics, metabolism, immune regulation and ecological resilience. This review examines how food systems may modulate gut microbiota and microbiome resilience within a One Health framework. Evidence from soil, crop and food microbiome studies suggests that local conditions and farming practices can leave detectable microbial signatures on plants and edible tissues. However, the soil–food–gut continuum should not be understood as a simple transfer route. Microorganisms and microbial products are repeatedly filtered by plant traits, farming systems, animal-production interfaces, harvesting, processing, storage, preparation and host physiology. The review also considers how this continuity may be weakened or redirected. Agricultural intensification, pollutants, post-harvest processing, antimicrobial use, ultra-processed foods, additive mixtures, low-fibre diets, early-life microbial disruption and reduced contact with environmental biodiversity may alter microbial communities at different points of the food system. Antimicrobial resistance is also discussed as a functional microbial trait that can circulate across human, animal, food and environmental interfaces. One Health approaches to food systems should therefore combine microbial risk control with microbial stewardship: protecting useful microbial diversity and function while preserving food safety. The aim is not to maximise microbial exposure, but to understand which microbial functions matter and how food systems can support gut microbiota resilience across environments, foods and hosts.

## 1. Introduction

Food systems can be understood as the set of elements and activities involved in the production, processing, distribution, preparation and consumption of food, together with their nutritional, health, socio-economic and environmental outcomes [1]. Food systems are central to human health and environmental sustainability, but they are also microbial systems. The way food is produced, processed, transported and consumed affects not only diet quality, soil health, biodiversity and ecosystem resilience, but also the microbial communities and exposures that connect environments, foods and hosts [2,3]. One Health is defined as an integrated and unifying approach that aims to sustainably balance and optimise the health of people, animals and ecosystems [4]. For this reason, the farm-to-fork pathway is an important space for One Health thinking, since it connects environmental, agricultural, animal and human health.

However, these systems are still often discussed mainly through nutrients, dietary patterns, food safety or environmental impact. These are essential dimensions, but they do not fully capture the microbial life that exists across the food system [5]. Soils, plants, animals, foods and the human gut all host complex microbial communities. These communities are involved in nutrient cycling, plant health, food characteristics, host metabolism and immune regulation [6]. They can also be affected by agricultural intensification, food processing, antimicrobial use, pollution and dietary simplification [7].

This review uses a microbiome-informed One Health lens to examine food systems as pathways of microbial exposure, ecological filtering and disruption. Instead of treating the gut microbiome as an isolated endpoint, it considers how upstream ecological conditions may influence microbial exposure, dietary substrates and microbiome resilience. The central idea is that food systems can support microbial continuity across soils, plants, foods and humans, but they can also contribute to microbial disruption when diversity, microbial functions or exposure pathways are weakened [6,7]. This disruption may occur through changes in environmental and food-associated microbiomes, but also through food processing, ultra-processed foods, antimicrobial pressure, food matrices and dietary practices. Here, microbiome resilience is understood as the capacity of microbial communities and functions to withstand disturbance, recover after disruption, or maintain ecological roles under changing environmental and dietary conditions.

The aim of this review is to provide a concise conceptual synthesis of how microbiomes connect food systems and One Health. It first discusses microbiomes as an under-integrated dimension of One Health. It then examines food as an ecological product within the soil–food–gut continuum, considers how modern food systems may disrupt this continuum, and finally outlines how microbiome-conscious food systems could help support microbial diversity, function and resilience. In doing so, it considers soil and crop management, food processing, dietary diversity, fibre-rich and minimally processed foods, legumes, fermented foods, food matrices, local food practices and microbial stewardship. Although animal-associated microbiomes are part of the wider One Health Microbiome, this review focuses mainly on the soil–plant–food–gut pathway, with animal systems discussed where they intersect with food production and antimicrobial resistance.

## 2. Literature Identification and Evidence Selection

This article was developed as a conceptual narrative review rather than a systematic review. The aim is not to provide an exhaustive quantitative synthesis, but to identify and integrate evidence that helps clarify how microbiomes connect food systems, dietary substrates, microbial exposure, environmental pressures and human health within a One Health framework.

Relevant literature was identified through targeted searches in PubMed/MEDLINE, Scopus, Web of Science and Google Scholar, complemented by backward and forward citation tracking of key papers. Searches focused primarily on peer-reviewed articles published between 2019 and 2026, in order to capture recent developments in microbiome science, food microbiology, soil ecology, antimicrobial resistance, ultra-processed foods, food additives, food matrices, fermented foods, microbiome-relevant dietary substrates, animal-production interfaces, climate-related food safety risks and One Health. Earlier studies were included when they were considered foundational for the argument, particularly where they provided seminal evidence on biodiversity exposure, obesity-related microbiota transfer, soil–plant microbial connectivity, environmental resistomes, early-life microbial assembly or diet-driven microbial loss.

Search terms were combined around six main thematic clusters: (i) One Health and microbiomes; (ii) soil, plant and edible food microbiomes; (iii) food processing, ultra-processed foods and food additives; (iv) environmental pollutants, pesticides, antimicrobial resistance and resistomes; (v) diet, dietary diversity, food matrices, fermented foods, early-life microbial exposure, biodiversity and gut microbiome resilience; and (vi) animal production, manure, aquaculture, climate change and food safety. Examples of search combinations included “One Health microbiome”, “soil plant gut microbiome axis”, “edible plant microbiome human gut”, “organic conventional fruit microbiome”, “ultra-processed food gut microbiota”, “food additives microbiome”, “food matrix gut microbiota”, “fermented foods gut microbiome intervention”, “legumes gut microbiota”, “dietary fibre carbohydrate-active enzymes microbiome”, “pesticides gut microbiota”, “antibiotic resistome soil food”, “livestock resistome manure soil”, “aquaculture antimicrobial resistance microbiome”, “climate change food safety microbiome”, “biodiversity exposure microbiota allergy” and “early life microbiome asthma”.

Priority was given to studies that contributed directly to the soil–plant–food–gut continuum or to mechanisms of microbial disruption along the farm-to-fork pathway. These included human cohort studies, controlled dietary interventions, metagenomic and food microbiome studies, agricultural microbiome studies, animal-production and resistome studies, mechanistic animal models, *in vitro* microbial screening studies, food safety and climate-related reviews, and high-quality reviews or perspectives. Studies were not selected solely on the basis of positive findings; particular attention was given to papers that clarified uncertainty, methodological limitations or the distinction between microbial exposure, transient passage, stable colonisation and functional impact.

Because this review addresses a broad interdisciplinary topic, the evidence was interpreted by strength and relevance rather than treated as equivalent. Human longitudinal studies, controlled interventions and large metagenomic analyses were considered particularly informative for diet–microbiome and exposure–microbiome links. Experimental models were used mainly to explore mechanisms. Agricultural, animal-production and food microbiome studies were used to support the ecological plausibility of microbial continuity across soil, plants, foods, animals and the gut. Studies on antimicrobial resistance were considered particularly relevant when they addressed resistance genes or resistant bacteria as mobile microbial functions across human, animal, food and environmental interfaces. Policy reports and institutional documents were used only to contextualise One Health, antimicrobial resistance, food safety and sustainable food systems, not as substitutes for primary scientific evidence.

## 3. Microbiomes as an Under-Integrated Dimension of One Health

One Health is commonly defined by the One Health High-Level Expert Panel as “an integrated, unifying approach that aims to sustainably balance and optimise the health of people, animals and ecosystems”. This definition, supported by FAO, WHO, WOAH and UNEP, is useful because it moves health away from a purely human-centred perspective and places it within the relationships between living organisms, environments and social systems [4]. One Health has often approached microorganisms through the problems they create: zoonotic diseases, antimicrobial resistance and the movement of pathogens across humans, animals and environments [7,8]. This focus remains essential, but it gives only a partial view of microbial life. Microbes are frequently treated as risks to be monitored and controlled, while their beneficial roles in maintaining ecosystems and host function receive less attention [7,9]. This distinction is important. Microbial risk control focuses on preventing harm caused by pathogens, contamination and antimicrobial resistance. Microbial stewardship adds a complementary perspective: it asks how beneficial microbial diversity, ecological functions and host-associated microbial relationships can be protected without compromising safety. In this sense, the aim is not to romanticise microbes or increase exposure indiscriminately, but to manage microbial life as part of the biological infrastructure that supports health.

Looking at this framework through microbiomes changes the emphasis. Microorganisms contribute to nutrient cycling, soil fertility, plant health, host metabolism, immune regulation and ecosystem resilience [6,10]. In the human host, microbial communities are distributed across several body sites, including the gut, oral cavity, skin, respiratory tract and urogenital tract, where they contribute to barrier integrity, immune signalling and metabolic homeostasis; when these communities are disturbed, they have been linked to diseases ranging from inflammatory and metabolic disorders to respiratory, cardiovascular and cancer-related conditions [11]. This is particularly clear in metabolic regulation: gut dysbiosis has been linked to altered glucose metabolism through several interacting pathways, including short-chain fatty acid production, bile acid metabolism, low-grade inflammation, intestinal permeability and changes in host energy balance [12]. These examples show why microbiomes should not be viewed only as microbial communities that accompany health or disease, but as functional systems that can influence host physiology, ecological stability and resilience. Health can therefore also be understood as an ecological condition shaped by exchanges between hosts, microbial communities and their surrounding environments [8,13]. Rather, it shows that One Health also needs to consider the microbial functions that support stability, adaptation and resilience.

This is the logic behind the One Health Microbiome. It describes microbiomes not as isolated communities, but as connected reservoirs of microbial genes, strains and functions that may be shared or exchanged across humans, animals, plants and environments [7]. These exchanges are shaped by contact, dispersal, ecological filtering and environmental conditions. Their consequences are still not fully understood, especially for commensal or beneficial microorganisms, but they show why microbiomes can be considered biological connectors across One Health domains [7].

Beneficial microorganisms also deserve clearer attention within this wider One Health perspective, but the terminology needs to be used carefully. Lactic acid bacteria provide a useful example because they operate across several One Health domains. In food systems, they contribute to fermentation, acidification and inhibition of spoilage or pathogenic microorganisms; in animal production, selected lactic acid bacteria and other probiotic strains may support gut microbial balance, immune function and disease resistance; and in humans, defined strains have been evaluated in specific clinical contexts. For example, *Bifidobacterium infantis* 35624 and *Saccharomyces boulardii* have been studied in irritable bowel syndrome and antibiotic-associated diarrhoea, respectively [14,15]. *Saccharomyces cerevisiae* var. *boulardii* CNCM I-1079 has been studied in neonatal piglets, while Bacillus-based probiotics have been explored in aquaculture and livestock for effects on gut microbiota, immune response, disease resistance and water quality [16,17]. In environmental and plant systems, related but distinct concepts are more appropriate: plant-growth-promoting rhizobacteria, rhizobia, mycorrhizae and microbial biocontrol agents may be used as microbial inoculants or biofertilisers to support nutrient acquisition, stress tolerance, plant health and pathogen suppression [18,19]. These examples show that beneficial microorganisms, including lactic acid bacteria and probiotics, are relevant across human, animal, food and environmental domains, but they should not be treated as interchangeable. The term “probiotic” should be reserved for live microorganisms that, when administered in adequate amounts, confer a health benefit on the host [20]. Fermented foods, probiotic strains, microbial metabolites, animal feed additives and plant-associated microbial inoculants all belong to the wider discussion of microbial stewardship, yet each requires its own evidence base, safety assessment and ecological context.

This perspective does not replace the surveillance of pathogens or antimicrobial resistance. It broadens One Health toward microbial stewardship: the protection of microbial diversity and functions that help sustain ecosystem stability and host resilience [9].

Food systems make this connection especially visible because they bring together several One Health domains in the same pathway. Soil management, crop production, animal husbandry, food processing, antimicrobial use, water quality, dietary patterns and consumer practices all influence microbial communities at different points of the farm-to-fork chain [18,21,22,23,24]. This makes food systems a particularly useful interface for studying microbiomes as biological connectors between environmental, plant, animal and human health [18,23]. From this perspective, individual diet remains central to gut microbiome modulation, but the ecological conditions that shape food production, microbial exposure and dietary substrates also deserve attention [6,25]. This includes not only the presence or absence of microorganisms in foods, but also wider conditions that shape microbial filtering, food matrices, antimicrobial pressure, dietary diversity and microbiome resilience across the food system.

## 4. From Soil to Gut: Food as an Ecological Product

Food is often described mainly through its nutritional composition: fibre, proteins, fats, minerals, vitamins and bioactive compounds [10]. This perspective is useful, but it can make food appear as a set of isolated components that act only after consumption. A microbiome perspective adds another layer. Food is also shaped by the environments, organisms and microbial communities involved in its production, processing, storage and preparation. In this sense, food can be understood as an ecological product, carrying not only nutrients, but also microbial substrates, metabolites, microorganisms and traces of its environmental history [5,8].

Soil is an important starting point for this continuum [13]. It harbours a large proportion of the planet’s biodiversity [3] and has been described as a microbial “seed bank” for plant-associated communities [6]. Evidence from grapevine systems illustrates this reservoir role. Soil was identified as a key source of vine-associated bacteria, while soil pH, carbon-to-nitrogen ratio and vineyard-specific properties helped shape microbial communities in roots, leaves and grapes [26]. Through the rhizosphere, phyllosphere and endosphere, plants interact with microbial communities that contribute to nutrient acquisition, stress tolerance and plant health [6,10]. These interactions may also influence the microbial and metabolic profile of edible plant tissues [25]. The microbial ecology of soil and plants is therefore not separate from the food system: it is part of the biological context in which food is produced.

This does not mean that microbes move directly or predictably from soil to the human gut. At each step of the food system pathway, microorganisms and microbial products may be selected, reduced, transformed or lost. These filters include plant species and plant tissues, agricultural practices, harvesting, washing, processing, storage, preparation and, finally, host physiology [7]. Still, soil and plant-associated microbiomes can shape the microbial exposure associated with food, especially in fresh fruits, vegetables and fermented products [25,27,28]. This is often discussed through the concept of the edible microbiome: the microbial communities associated with edible plant tissues and food products. A study comparing the microbiota of organically and conventionally managed apples provides a concrete example. Bacterial communities were detected across peel, pulp, seeds, stem and calyx. The authors found that organic apples had higher bacterial diversity overall, with the pulp emerging as a particularly diverse compartment, whereas conventional apples showed the highest diversity mainly on the peel [29]. Food can therefore act as a biological bridge between environmental microbial communities and the gut ecosystem. This is also why comparisons between the rhizosphere and the gastrointestinal tract are useful: both are microbe-rich interfaces where nutrient exchange, host–microbe interactions and ecological filtering take place [6,10].

This edible microbiome perspective makes the soil–food–gut connection more concrete. Fruits, vegetables and fermented foods can carry live microbial communities shaped by the conditions they encounter before consumption [25,27,28]. Fermented foods are a particularly relevant example, because they may contain lactic acid bacteria and other food-associated microorganisms; however, these exposures should be distinguished from probiotic strains, which require strain-level characterisation and evidence of host benefit [20,22]. Some food-associated microbes can survive gastrointestinal transit and may contribute to microbial exposure in the gut [5]. However, survival through the gut does not necessarily mean stable colonisation, and the long-term relevance of these microbes for gut microbiome composition and host health is still not fully understood [6,25]. For this reason, food-associated microbes should be understood as part of the broader microbial exposure created by diet and food systems, rather than simply as permanent members of the gut microbiome [5].

This reframes the farm-to-fork pathway as more than a logistical chain. Food connects upstream ecological conditions with downstream human microbial exposure through nutrients, fibres, metabolites, microbial communities and environmental signals [6]. This continuity is central to a One Health view of food systems because it links soil, plant, food and human health within the same ecological frame [7,9].

At the same time, this pathway is not linear. Microbial movement across the food system is shaped by selection, loss, transformation and filtering at each stage [7]. Farming practices, processing, storage, cooking and host physiology all influence which microorganisms or microbial products persist, disappear or become biologically relevant [25,30]. These same stages can also reshape food matrices and microbial substrates, influencing not only microbial exposure but also the conditions under which food components become available for microbial metabolism. This is why the health relevance of food cannot be separated from the microbial and environmental conditions that produced it [6].

## 5. Disrupting the Continuum: Modern Food Systems and Microbiome Resilience

If food systems can support microbial continuity, they can also interrupt it. The soil–food–gut connection described in the previous section depends on ecological conditions such as soil biodiversity, plant–microbe interactions, food-associated microbial communities and the availability of dietary substrates [6,7]. Modern food systems may disturb these conditions at different stages of the farm-to-fork pathway, altering microbial diversity, exposure and function before food even reaches the gut [25]. Rather than representing a single break in the chain, these disruptions occur through several ecological filters: agricultural management, chemical exposure, post-harvest processing, dietary patterns, animal-production interfaces, early-life microbial assembly and antimicrobial pressure. Each filter can reduce, select, transform or redirect microbial communities and functions along the soil–food–gut continuum.

### 5.1. Agricultural Management and Chemical Pressure

At the agricultural level, intensive production practices, including reduced crop diversity, intensive tillage, high fertilisation and repeated chemical inputs, may alter microbial community composition and function, reduce soil biodiversity, disturb microbial interaction networks and weaken the functional redundancy that allows soils to maintain key functions under environmental stress [3]. Antimicrobial residues and antimicrobial use in agroecosystems add another layer of concern, since they may contribute to the dissemination of antimicrobial-resistant bacteria and resistance genes while also affecting non-target microbial communities in soil and plant-associated environments. Aminoglycosides, tetracyclines and quinolones are among the antibiotic classes commonly used in plant production that are also critically important for the treatment of bacterial infections in both animals and humans [31].

Apple orchard studies show that management effects are especially visible in soil fungi: integrated pest management orchards had lower fungal diversity indices and distinct fungal communities compared with organic orchards, although bacterial diversity and functional groups were less strongly affected [32]. Similar evidence from Gannan navel orange orchards suggests that organic management can increase soil bacterial alpha-diversity, microbial functional diversity and network complexity compared with conventional farming, indicating that farming systems can reshape not only microbial composition but also potential ecosystem function [33]. This matters because soil microorganisms are involved in nutrient cycling, organic matter decomposition, plant nutrient acquisition and stress tolerance. Agrochemicals and emerging pollutants, including microplastics, may also act as selective or cumulative pressures on microbial communities, although their effects depend on the compound, dose, microbial taxa and environmental context [3]. Together, these pressures can weaken the microbial reservoir from which plant-associated communities are recruited and may influence the microbial and biochemical profile of edible crops [23]. They also raise a broader question: whether chemicals used or released across food systems may act as selective pressures beyond the field, including on microbial communities associated with foods and the human gut. This concern is therefore not limited to soil microbiomes. A systematic *in vitro* screen of 1,076 industrial and agricultural chemicals against 22 common human gut bacterial species identified 168 chemicals with inhibitory activity, with fungicides and industrial chemicals among the groups showing the strongest anti-gut-bacterial effects [34]. Pesticide-focused work adds a mechanistic layer to this concern, showing that some pesticides can either inhibit or promote the growth of particular gut microbial species and may accumulate within microbial or host-associated systems, potentially prolonging their biological effects [35]. These examples suggest that disruption can begin before harvest, at the level of soil management, crop exposure and chemical pressure, but may extend downstream through food-associated exposures and gut microbial responses.

### 5.2. Post-Harvest Processing

Yet the microbial profile of food continues to be reshaped after production, as foods move through harvesting, washing, storage, transport, preservation, processing and preparation. These steps are essential for food safety, shelf life and distribution, especially in large-scale food systems, but they can also reshape the microbial communities associated with food before consumption [25,30].

Consumers often associate microbial presence in foods with spoilage or contamination. Food technologies have therefore developed to reduce microbial contamination and extend shelf life. This does not mean that processing is inherently harmful. One of the oldest methods of food transformation to enhance food shelf life and safety is fermentation, which can enrich foods with live microorganisms and microbial metabolites, although it may also select for a smaller number of dominant taxa compared with raw materials [5,28]. Historically, this role is illustrated by products such as *narezushi*, an early fermented fish preparation in which salting and fermentation with rice helped preserve fish and improve food stability [36,37]. However, fermented foods should not be understood only as preservation strategies; they are also food matrices shaped by microbial activity. Their microbial composition depends on the raw material, spontaneous or starter-driven fermentation, processing conditions, storage, and whether the final product is consumed live or after heat treatment [22,38].

This distinction is important because fermented foods are highly diverse. Yoghurt, kefir, cheeses, fermented vegetables, kombucha and other fermented products may contain lactic acid bacteria, acetic acid bacteria, yeasts or other microbial groups, but their effects cannot be generalised simply because they are “fermented”. Lactic acid bacteria are central to many familiar fermented foods, including yoghurts, cheeses and kefir, where they contribute not only to preservation and shelf life, but also to acidification, texture, flavour development and the production of microbial metabolites. Large-scale production often relies on selected microbial cultures, which improves safety, reproducibility and sensory consistency, but may also reduce the microbial complexity found in some traditional or spontaneous fermentations. The influence of fermented foods on the gut microbiota therefore depends on the strains present, their viability at consumption, the food matrix, the metabolites produced during fermentation, the dose and frequency of intake, and the background diet of the consumer [22,38].

Human intervention studies support the view that fermented foods can modulate the gut microbiota, although effects vary by product and host context. In the randomised dietary intervention by Wastyk et al. [39], a high-fermented-food diet steadily increased gut microbiota alpha-diversity and reduced several inflammatory markers, whereas the high-fibre arm mainly increased microbiome-encoded carbohydrate-active enzymes and showed more individualised immune responses. More recently, Schropp et al. [40] used a crossover intervention design to examine regular sauerkraut consumption and showed that daily intake of fresh or pasteurised sauerkraut for four weeks altered gut microbial composition and metabolomic profiles in healthy adults. The comparison between fresh and pasteurised sauerkraut is particularly informative because it suggests that fermented foods may act not only through live microorganisms, but also through the food matrix, fermentation-derived metabolites and postbiotic components [40].

Kombucha provides a useful example of this complexity. It is produced by fermenting sweetened tea from *Camellia sinensis* with a symbiotic culture of bacteria and yeasts, commonly referred to as SCOBY, and its composition is influenced by the tea substrate, sugar source and fermentation conditions [41,42]. Current human evidence remains limited: a small randomised crossover pilot study in adults with diabetes reported lower fasting blood glucose after four weeks of kombucha intake, but the sample size was small and the findings require cautious interpretation [43]. Kombucha is nevertheless useful conceptually because comparisons between live kombucha, pasteurised kombucha and non-fermented controls can help separate the contribution of viable microorganisms from that of the fermented matrix and its metabolites [42,43].

Cheese is another useful example of why fermented foods should be discussed as complex matrices rather than as simple sources of saturated fat, salt or live microbes. A recent review argues that cheese contains a structured dairy matrix with microbial communities, peptides, minerals, polar lipids and fermentation-derived compounds that may influence cardiometabolic outcomes in ways that are not fully predicted by its saturated fat content alone [44]. In the present review, this is relevant because fermented foods bring together microbial exposure, food structure, dietary substrates and safety considerations within the same food-system interface.

Pasteurisation, described for the first time by Louis Pasteur in 1864, provides a contrasting example of microbial management. By applying controlled heat treatments, it reduces microbial contamination and improves food safety and shelf life, but it also changes the viable microbial exposure associated with foods such as milk, juices and other beverages. Raw products are sometimes perceived as having better nutritional or sensory properties than pasteurised products, but they can also carry higher food-safety risks. However, this claim depends on the product and the outcome being considered. Macdonald et al.’s [45] meta-analysis clearly shows that pasteurisation has a minimal impact on the nutritional value compared to raw milk (unpasteurized). For raw milk, available evidence suggests that potential nutritional differences do not outweigh the increased microbiological risk. In other food products, such as fruit juices, degradation of vitamins is real and measurable, which is why nonthermal pasteurisation techniques are being widely used, such as pulsed electric fields and high-pressure processing or pascalization [46].

In fruits, washing is commonly used to reduce microbial surface contamination. However, resident microbial communities may also contribute to protection by competing with spoilage organisms or pathogens [47,48]. However, post-harvest handling must balance the possible protective role of resident microbiota with the need to reduce pathogens and spoilage organisms. Pre-harvest factors such as monoculture and varietal uniformity may also influence susceptibility to pathogen spread and spoilage, linking agricultural management with post-harvest microbial risk [49,50,51]. Therefore, chemical treatments, such as antifungal treatments, are applied to reduce fruit spoilage during storage.

Fruit conservation is normally done by cold storage, a method very effective to prolong fruit quality [52]. Other techniques can be added, such as controlled atmospheres, consisting of changing the composition of nitrogen, oxygen and carbon dioxide, leading to a reduced fruit respiration rate (lowering fruit decay process) and limiting microbial growth (reducing fruit spoilage). These techniques do not necessarily remove microbial presence from stored fruit, but may select for microorganisms able to persist under lower temperatures, reduced oxygen and higher carbon dioxide conditions [30,53,54]. Therefore, post-harvest storage does not eliminate microbial life; it can also select for microbial communities adapted to specific storage environments.

The key point is that post-harvest practices do not simply preserve food; they also influence which microbial exposures are retained, reduced, transformed or standardised before reaching consumers [5,24]. From a microbiome-conscious perspective, the challenge is therefore not to oppose processing, but to understand how different post-harvest practices balance food safety, shelf life, food quality and microbial exposure.

### 5.3. Diet, Ultra-Processed Foods and Additives

The next filter is the diet itself. Even if food-associated microbes and metabolites reach the consumer, their relevance for the gut ecosystem depends on the wider dietary pattern, including fibre availability, plant diversity, food structure and repeated exposure to processed products. Urbanised lifestyles and diets high in ultra-processed foods may reduce both environmental microbial exposure and the diversity of substrates available to gut microbes [2,10,24]. This dietary shift is also associated with wider health concerns. An umbrella review including 45 pooled analyses and almost 9.9 million participants found that higher ultra-processed food exposure was associated with a higher risk of several adverse outcomes, particularly cardiometabolic, mental health and mortality-related outcomes [55]. From a food-systems perspective, this supports the argument that ultra-processed foods are not only individual products, but part of a dietary pattern that displaces meals based on fresh or minimally processed foods and culinary preparations, thereby worsening diet quality and contributing to the burden of diet-related chronic disease [56]. Large-scale human data reinforce the link between diet and microbial ecology. In 10,068 participants from the Human Phenotype Project, app-based dietary records combined with shotgun metagenomics showed that diet predicted microbial richness and diversity, the relative abundance of 669 out of 724 microbial species tested, and 313 out of 320 microbial pathways. Specific food–microbe associations were also identified, including coffee with *Lawsonibacter asaccharolyticus*, yoghurt with *Streptococcus thermophilus* and milk with *Bifidobacterium* species, while the degree of food processing emerged as an important predictor of microbiome composition [57]. These diet–microbiome associations matter because microbial shifts may not always be passive markers of dietary intake. A landmark experiment using germ-free mice showed that gut microbiota transferred from human twin pairs discordant for obesity partly reproduced the donor phenotype: mice receiving microbiota from the obese twin gained more fat than those receiving microbiota from the lean twin. However, the study also showed that this effect depended on ecological context. When mice carrying obese-associated microbiota were housed with mice carrying lean-associated microbiota, lean-associated microbes could invade the obese-associated community and reduce fat gain, but this protective effect was favoured by a diet low in saturated fat and rich in fruit and vegetables. Under a less favourable diet, microbial invasion was limited. This study therefore illustrates a bidirectional ecological axis: microbiota can shape host metabolism, while diet and microbial community interactions shape what the microbiota can do [58].

Food additives may represent an additional layer of exposure within ultra-processed diets. In the NutriNet-Santé cohort, higher exposure to food colouring additives as a group was associated with a 38% higher risk of type 2 diabetes; among individual additives, ordinary caramel (E150a) and curcumin (E100) were associated with 46% and 49% higher risks, respectively [59]. Preservatives may also be relevant in this discussion. In NutriNet-Santé, higher exposure to preservatives as a group was associated with a 24% higher risk of hypertension; non-antioxidant preservatives were associated with a 29% higher risk of hypertension and a 16% higher risk of cardiovascular disease, while potassium sorbate (E202) was individually associated with a 39% higher risk of hypertension [60]. Importantly, additive exposure rarely occurs one compound at a time. In NutriNet-Santé, two widely consumed food additive mixtures were positively associated with type 2 diabetes incidence, suggesting that mixture-based approaches may better reflect real-life ultra-processed dietary exposure than single-additive assessments [61]. These findings remain observational and should not be interpreted as proof of causality. Nevertheless, they support the need to evaluate ultra-processed foods as complex exposure matrices involving altered food structure, low fibre availability, additive mixtures and possible microbiome-relevant effects. Although these cohort findings do not establish direct microbiome-mediated mechanisms, they reinforce the idea that ultra-processed diets should be considered as combined nutritional, structural and chemical exposures rather than as isolated ingredients.

Several microbiome-relevant mechanisms may help explain why ultra-processed dietary patterns deserve attention beyond their association with chronic disease outcomes. First, these diets often displace fibre-rich minimally processed foods, reducing the availability of microbiota-accessible carbohydrates that support saccharolytic fermentation and short-chain fatty acid production; experimental work in humanised mice has shown that low-fibre diets can deplete gut microbial diversity, with losses becoming harder to reverse across generations when missing taxa are not reintroduced [24,62]. Second, the physical structure of ultra-processed foods may alter digestion kinetics, nutrient release and the delivery of substrates to the colon; this is consistent with the broader argument that ultra-processed foods act not only through nutrient composition, but also through altered food matrices, formulation and replacement of minimally processed foods [63]. Third, additives may interact with gut microbial communities directly or indirectly. In mice, two widely used emulsifiers, carboxymethylcellulose and polysorbate-80, altered gut microbiota composition, promoted low-grade inflammation and contributed to metabolic syndrome-like features [64]. Non-nutritive sweeteners also provide mechanistic evidence: early work showed that saccharin, sucralose and aspartame could induce glucose intolerance through microbiota-dependent mechanisms [65], while a later human intervention study showed personalised microbiome-driven effects of non-nutritive sweeteners on glycaemic responses [66]. Fourth, ultra-processed foods are usually consumed as part of wider dietary patterns that combine low fibre, high energy density, altered food matrices and repeated exposure to additive mixtures. This is consistent with large-scale human data showing that diet, individual foods and degree of processing are associated with gut microbiome composition, diversity and functional potential [57], and with NutriNet-Santé analyses suggesting that additive mixtures may better reflect real-life exposure than single compounds [61]. The microbiome relevance of ultra-processed foods should therefore be framed cautiously: the concern is not a single ingredient or a single microbial pathway, but the combined effect of reduced microbiota-accessible substrates, altered food structure and repeated chemical exposure.

### 5.4. Early-Life Microbial Assembly

The consequences of reduced microbial exposure may be especially relevant early in life, when the infant gut microbiome is still being assembled and is sensitive to delivery mode, breastfeeding, antibiotics, diet, household contacts and environmental exposure [7,25]. Rather than acting as isolated variables, these exposures form a sequence of ecological filters that influence which microorganisms arrive, persist, compete or disappear during early colonisation.

Within this sequence, delivery mode is one of the earliest and most studied determinants. Caesarean delivery has been linked to reduced early microbial diversity, delayed colonisation by Bacteroidetes/Bacteroides and altered immune-related trajectories, although these associations are shaped by additional factors such as antibiotic exposure and breastfeeding [67]. A large neonatal microbiome study provided an important example of this disruption: infants born by caesarean section showed reduced transmission of maternal Bacteroides strains and higher colonisation by hospital-associated opportunistic pathogens, including *Enterococcus*, *Enterobacter* and *Klebsiella* species [68]. These studies show that birth mode matters, but they also point to a broader issue: early colonisation is not a single event at birth, but a dynamic process shaped by repeated microbial inputs and by the infant gut environment itself.

Recent infant microbiome data from the United States suggest that early-life microbial disruption is not limited to delivery mode alone. In a cohort of 412 infants, 24% had no detectable *Bifidobacterium*, including both vaginally and caesarean-born infants. The authors suggest that this pattern may reflect failed or insufficient acquisition of infant-associated *Bifidobacterium* from early environmental and caregiving sources, together with priority effects in which other human-milk-oligosaccharide consumers occupy the same ecological niche first. In caesarean-born infants, this niche was more often occupied by potentially pathogenic species such as *Clostridium perfringens*, supporting the idea that early microbial assembly depends on both microbial acquisition and ecological niche occupation [69]. This points to a broader feature of early-life microbiome assembly: it depends not only on birth and feeding, but also on whether the surrounding environment provides repeated opportunities for microbial acquisition and ecological replacement.

In industrialised settings, reduced contact with diverse environmental microbiomes has been linked to altered colonisation patterns, although these relationships are complex and should not be reduced to a single cause [7,24]. This is consistent with the biodiversity hypothesis, which proposes that reduced contact with natural environmental biodiversity may impair the development of diverse commensal microbiota and weaken immunoregulatory capacity. In Finnish adolescents, lower environmental biodiversity around the home was associated with altered skin microbiota and higher atopy prevalence [70]. Evidence from farm-exposed children also supports this link: in the PASTURE/EFRAIM cohorts, early-life farm exposure was associated with more mature gut microbiome development, and this maturation partly explained the protective farm effect against childhood asthma [71]. A small intervention study in Finnish daycare centres also suggests that this type of microbial exposure can be partially restored in urban settings as well. Replacing standard yard surfaces with forest floor, lawn and planter boxes for 28 days increased children’s contact with environmental biodiversity and was associated with shifts in skin and gut microbiota, together with changes in immune markers consistent with enhanced immunoregulatory activity [72].

These studies are not food system studies in a narrow sense, but they are useful for interpreting the food system continuum within a wider One Health ecology. They show that microbial exposure is shaped by the combined effects of diet, built environments, land use and contact with biodiverse ecosystems. The concern, therefore, is not only nutritional composition. It is also the gradual narrowing of microbial and ecological inputs that help shape gut microbiome development and resilience [6].

Experimental work on mice colonised with human microbiota showed that diets low in microbiota-accessible carbohydrates depleted gut microbial diversity; within one generation, many changes were reversible when fibre was restored, but across successive generations some microbial losses became difficult to recover without reintroducing the missing taxa [62]. This supports the idea that dietary substrates, especially fibre and other microbiota-accessible carbohydrates, are not only short-term modulators of the gut microbiome but may also influence resilience and recovery after disruption. Microbial disruption is therefore not only a matter of diversity loss. It may also involve the selection, persistence or spread of microbial traits that are undesirable from a One Health perspective.

### 5.5. Antimicrobial Resistance and Animal-Production Interfaces

Unlike many microbiome disruptions discussed in this review, antimicrobial resistance (AMR) is not only a change in microbial composition. It also involves the emergence, persistence and circulation of a functional genetic reservoir: the resistome. The resistome encompasses the AMR genes present in microbial communities, many of which may be located on mobile genetic elements that can be exchanged between taxa and environments. In this sense, AMR should be understood as a property of ecological networks, and not only of isolated pathogens or clinical environments [73,74].

Antibiotic exposure illustrates this point at the level of the human gut. In a population-based study of 14,979 individuals, oral antibiotic use during the eight years before faecal sampling was associated with differences in gut microbiome composition, with the strongest associations after recent exposure but detectable signatures even several years later [75]. These findings reinforce the idea that antimicrobial pressure can leave long-lasting ecological traces within host-associated microbial communities.

Within a One Health framework, the resistome forms a connected and dynamic system encompassing human clinical settings, animal production systems, agricultural soils, aquatic ecosystems and food chains. Horizontal gene transfer, environmental contamination and anthropogenic selective pressures allow resistance determinants to move and persist between these compartments. For this reason, AMR cannot be fully interpreted solely within the boundaries of hospitals or animal production systems; it should also be considered as an ecological and evolutionary phenomenon embedded in interconnected microbial ecosystems [76,77,78].

The selective pressures driving the expansion of the resistome extend beyond the therapeutic use of antibiotics in human medicine. Veterinary and agricultural applications of antimicrobials, aquaculture practices and environmental exposure to antimicrobial residues can contribute to the maintenance and enrichment of resistance genes in environmental reservoirs [76,79]. Non-antibiotic compounds, including biocides, heavy metals and certain agrochemicals, may also co-select AMR through interconnected genetic mechanisms, reinforcing the stability and persistence of resistance determinants in microbial communities [80,81].

Animal production is a central interface in this process. Antibiotics remain important for treating bacterial infections and protecting animal welfare, but inappropriate or excessive use in livestock can select for resistant bacteria and resistance genes within animal gut microbiomes, manure and farm environments. Antimicrobial stewardship in animal production generally prioritises therapeutic use, restricts prophylactic use to exceptional circumstances, and rejects use as growth promoters [82]. According to the Ninth Annual Report of the World Organisation for Animal Health, 71% of the 157 participating countries reported no longer using antimicrobials for growth promotion in animals; however, 22% still continued this practice, highlighting the persistent global challenge of AMR [83]. Globally, antimicrobial use in livestock reached approximately 110,777 t in 2019 and could increase by nearly 30% by 2040 without significant intervention [84].

Production intensity may influence AMR dynamics, but the relationship is not straightforward. High-density animal-production systems may increase disease pressure, concentrate waste streams and favour preventive or group-level antimicrobial use when biosecurity, welfare or management conditions are poor [85,86]. Extensive or pasture-based systems may reduce some density-related pressures, but they may involve greater contact with soil, water, wildlife and diffuse manure deposition [87,88,89]. The microbiome and AMR implications therefore depend not only on whether a system is described as intensive or extensive, but on antimicrobial stewardship, animal health management, biosecurity, stocking density, waste treatment, water quality and environmental safeguards, all of which influence whether animal-production systems amplify or limit the circulation of resistant bacteria and resistance genes across food-system interfaces [76,77,78,90].

A meta-analysis examining AMR in food-producing animals between 2000 and 2018 provides a useful comparison across livestock species. The study assessed resistance patterns in cattle, swine and poultry and reported that the proportion of AMR increased over time in all three groups: from 12% to 23% in cattle, from 13% to 34% in swine and from 15% to 41% in poultry. Among the bacterial taxa assessed, the highest resistance rates in *Campylobacter* spp. were reported for tetracyclines and quinolones, both around 60%. In *Escherichia coli*, quinolone and gentamicin resistance ranged between 20% and 60%, while in *Salmonella* spp. resistance ranged from 5% to 38%. In *Staphylococcus aureus*, the highest resistance rates were associated with penicillins, ranging from 40% to 80% [91]. Poultry-production systems have also been identified as important reservoirs of antimicrobial-resistant bacteria, including multidrug-resistant *Escherichia coli* [92]. Resistance to tetracyclines, sulfonamides and β-lactam antibiotics has been frequently reported in intensive poultry-production and swine-production environments, where antimicrobials administered through feed or water may contribute to the selection and dissemination of resistant microorganisms [92,93].

Manure is one of the clearest material links between animal and crop systems. When recycled as fertiliser, it can support soil organic matter and nutrient cycling, but it may also introduce antimicrobial residues, resistant bacteria and resistance genes into soil–plant environments if not properly treated or managed. Other interfaces can further facilitate resistome circulation throughout the food system. Hospital and community wastewater systems can introduce clinically relevant resistance genes into environmental microbial communities [94]. In agricultural settings, treated animal manure and by-products can contribute to soil resistomes, which may in turn interact with plant-associated microbiomes [90]. Irrigation water represents another point of convergence linking the environmental and agricultural compartments. Food products may also act as transient vectors of resistant bacteria or resistance genes, allowing indirect exposure of the human gut microbiome to environmental resistomes through diet [95,96].

Aquaculture practices should also be considered among the selective pressures that can contribute to the maintenance and enrichment of resistance genes in environmental reservoirs, adding another route through which the food-system resistome can connect aquatic environments, food products and human exposure [76,79].

The overuse and misuse of antibiotics can therefore contribute to environmental contamination through resistant microorganisms, antimicrobial residues and contaminated by-products, including food products, water sources and animal waste applied to agricultural soils. This is particularly important because several antimicrobial classes used in animal production are also relevant in human healthcare [93]. The scale of the AMR problem is substantial. In 2019, AMR was estimated to be directly responsible for 1.27 million deaths worldwide and associated with 4.95 million deaths [97]. These data should not be interpreted as evidence that animal production is the only or main driver of AMR, but they underline its importance as one of several One Health interfaces where resistant bacteria and resistance genes may be amplified or redistributed.

AMR is therefore not limited to a clinical problem of treatment failure, but represents an ecological and evolutionary phenomenon embedded in One Health systems. Combating AMR requires integrated management strategies that consider not only antimicrobial use in human and veterinary medicine, but also environmental management, agricultural practices, food production systems, manure and wastewater treatment. Surveillance approaches should also go beyond clinical isolates and include environmental, agricultural and food-related resistomes in order to capture the wider ecological scope of resistance gene circulation.

Ultimately, AMR illustrates how functional microbial traits can spread through the same interconnected systems that underpin microbial diversity and ecological resilience. In a microbiome-informed One Health approach, the challenge is twofold: to preserve beneficial microbial functions in different environments while limiting the amplification and dissemination of resistance genes within those same interconnected ecological networks.

### 5.6. Climate and Environmental Pressures

Broader environmental pressures further complicate this continuum. Climate change, water stress, land-use change and biodiversity loss all influence agricultural systems, including the microbial communities that support soil and plant health [3]. These links should not be overstated, because the relationships between environmental change, food-system microbiomes and gut resilience are still being clarified. Even so, they point in the same direction: microbial disruption is not limited to the gut. It can begin upstream, through changes in soil management, agricultural chemicals, food processing, dietary simplification, early-life environments and antimicrobial pressure. These pressures do not act independently; they interact across the farm-to-fork pathway, shaping the microbial diversity, functions and exposures that eventually reach the consumer [6]. 

Soil microorganisms provide a variety of important ecosystem services [98], including nutrient cycling [99,100], organic matter decomposition [101], carbon sequestration [102], plant productivity [103], disease suppression [104] and soil aggregation [105]. 

These soil microbial communities form the foundation of broader food-system microbiomes, influencing the microorganisms associated with crops, livestock, agricultural environments and, ultimately, food. However, they can be highly sensitive to climate change and pollution [106,107]. The increase in temperature, especially, has been observed to have an impact on soil microorganisms, affecting their growth [108], diversity [109], and functions, such as their ability to decompose organic matter [110] and nutrient transformation [111]. 

Furthermore, agricultural intensification and land-use changes can amplify these environmental pressures. Monocultures, intensive tillage and long-term use of fertilisers and pesticides have been associated with changes in soil microbiomes diversity, their network complexity and functional redundancy [112,113,114]. In one study, for example, it was found that the long-term continuous tomato monoculture altered nutrient availability and soil pH, leading to significant shifts in the rhizosphere bacterial composition and their functions [115]. In contrast, diversified farming systems incorporating crop rotations, cover crops, agroecological practices and reduced chemical inputs often support richer and more stable microbial communities [116,117,118]. 

Extreme weather events such as floods, storms and prolonged droughts may further redistribute microorganisms, sediments, nutrients and contaminants across landscapes, altering microbial community assembly and disrupting established ecological relationships [119,120,121]. 

The effects of climate and environmental change extend beyond soil. Plant microbiomes are shaped by interactions between soil, climatic conditions and host physiology, with water representing a particularly important pathway linking microbial ecology and food production—changes in water availability, quality and reuse practices can affect microbial communities and pathogen transmission simultaneously. For example, water stress can substantially alter rhizosphere microbial assemblages, affecting nutrient acquisition, stress tolerance and resistance to pathogens [122,123]. 

Rising temperatures, altered seasonality and shifting climatic zones can also facilitate the expansion of insect pests, plant pathogens and invasive species into new regions, potentially increasing crop losses and the demand for pesticide applications [124,125]. While pesticides may provide short-term crop protection, increased reliance on chemical control can have unintended consequences for non-target microbial communities, contributing to reductions in diversity and alterations in microbial functions. Importantly, climatic pressures may simultaneously favour certain pathogenic microorganisms while reducing populations of beneficial taxa that support ecosystem functioning and crop resilience [126,127].

Beyond soil and plant microbiomes, climate changes can also influence the survival, growth and transmission of foodborne bacterial pathogens [128]. Warmer environmental conditions have been associated with shifts in the prevalence and geographical distribution of pathogens such as *Salmonella enterica*, *Campylobacter jejuni* and pathogenic *Vibrio* species, some of which are expanding into regions where they were previously uncommon [128,129,130,131]. Heavy rainfall and flooding can also facilitate the transport of microbial contaminants from livestock operations, wildlife reservoirs and wastewater systems into agricultural soils and irrigation water, increasing contamination risks for fresh fruits and vegetables consumed raw [95,131,132]. Conversely, drought conditions may reduce the availability of high-quality irrigation water and increase reliance on alternative water sources while concentrating biological and chemical contaminants in limited water supplies [95,133,134]. Furthermore, climate-related disruptions may also affect food storage, transport and cold-chain infrastructure, potentially increasing microbial spoilage and pathogen proliferation during post-harvest handling, particularly during heatwaves and extreme weather events [128,131,135].

Beyond bacterial pathogens, climate change may likewise impact the occurrence of fungal pathogens. Temperature and humidity strongly regulate the growth and toxin production of species belonging to the genera *Aspergillus*, *Fusarium* and *Penicillium* [136,137,138]. As climatic conditions become more favourable for these organisms in some regions, concerns have emerged regarding the expansion of aflatoxins, fumonisins and other mycotoxins into areas historically considered low-risk [136,137,138,139]. Such risks are particularly relevant for cereals, maize, nuts and dried fruits, which are highly susceptible to fungal contamination during cultivation and storage [136,137,138].

Similar processes are occurring in aquatic food systems, where rising water temperatures, altered nutrient cycling and more frequent extreme weather events can promote harmful algal blooms and the accumulation of marine biotoxins, affecting shellfish, fish and other seafood products [140,141,142].

Consequently, climate-related microbial risks are not distributed uniformly across food systems: fresh fruits and vegetables are particularly vulnerable to contamination through irrigation water and flooding events, cereals and nuts are especially susceptible to mycotoxin-producing fungi, other seafood products, especially shellfish, are disproportionately affected by marine pathogens, harmful algal blooms and biotoxin accumulation. In parallel, changing environmental conditions may also influence the ecology and dissemination of AMR genes across agricultural and aquatic environments, further challenging food safety and public health [76,143,144,145]. 

Taken together, current evidence suggests that climate change and environmental pressures, together with increasing agricultural intensification, are reshaping microbial diversity, functions, and exposure pathways throughout the farm-to-fork continuum, by influencing which beneficial and pathogenic microorganisms persist in soils, colonise plants, survive processing and storage, and ultimately reach consumers. In this sense, climate change modifies the ecological filters that govern microbial transmission from environment to food, and from food to humans, altering both the risks and benefits associated with microbial exposure. Understanding these interconnected processes is, therefore, increasingly important for developing food systems that are productive, resilient and safe at the same time, while also maintaining the microbial diversity which both ecosystems and human health depend upon.

## 6. Toward Microbiome-Conscious Food Systems

If modern food systems can weaken microbial continuity, the response cannot focus only on the human gut. It also needs to consider what happens earlier in the farm-to-fork pathway: how soils are managed, how crops are produced, how foods are processed, and how dietary patterns shape microbial exposure [10]. A microbiome-conscious food system would therefore aim to protect the conditions that allow useful microbial diversity and function to persist across soils, foods and hosts [7,9]. In practical terms, this means beginning to evaluate food-system practices not only by yield, safety, nutrient profile or emissions, but also by how they affect microbial diversity, exposure and ecological function. Table 1 summarises examples of intervention levels where microbiome-conscious food systems could be assessed, linking practical actions with microbiome-related and health or environmental indicators.

At the agricultural level, this starts with soil health [6,23]. Practices such as crop rotation, cover cropping, organic amendments and reduced tillage may help maintain soil microbial diversity and the functions linked to nutrient cycling, plant health and stress tolerance [3,6]. Evidence from organic citrus orchards suggests that farming systems can increase soil bacterial alpha-diversity, functional diversity and microbial network complexity, supporting the view that microbiome-conscious agriculture should focus on habitat conditions and soil management rather than isolated microbial inputs alone [33]. Orchard studies also show that management type can alter below-ground microbial ecosystems in different ways: fungal communities were more sensitive to management than bacterial communities, and organic orchards showed higher fungal richness and phylogenetic diversity than IPM orchards [32]. Biofertilisers and microbial consortia may also be useful in some contexts, but they should not be treated as simple technological fixes. Their effectiveness depends on local soil, crop, climate and management conditions [3]. In this sense, microbiome-conscious agriculture is less about adding isolated microbes and more about creating conditions where beneficial microbial communities can function [9].

Diet is another part of the same continuum. The planetary health diet proposed by the EAT–Lancet Commission is useful here because it connects human nutrition with environmental sustainability, especially through more diverse and plant-rich dietary patterns [2]. For this review, its relevance is not only nutritional or environmental, but also ecological. A greater diversity of plant foods can provide a wider range of fibres, polyphenols and other microbial-accessible substrates, which may support a more diverse and metabolically flexible gut microbiome [6,24]. Within this diversity, fibre-rich minimally processed foods deserve particular attention because they provide microbiota-accessible substrates within more complex food matrices [24,146]. This view is supported by large-scale diet–microbiome analyses showing that plant-food diversity, specific foods and overall dietary patterns are associated with microbial composition and functional potential, suggesting that dietary diversity can be treated as an ecological input to the gut microbiome rather than only as a nutritional variable [57]. Within this broader pattern, legumes are especially relevant because they combine plant protein with fermentable fibres, resistant starch and phytochemicals, linking dietary quality, sustainability and microbial metabolism [147]. Recent large-scale diet–microbiome evidence also identified pulses and their products among dietary features associated with microbial species abundance and functional pathways, while intervention evidence suggests that high-fibre dietary patterns, including legumes, can increase microbiome-encoded carbohydrate-active enzymes [39,57].

This also means that microbiome-conscious food systems should consider not only the inclusion of beneficial foods, but also the displacement effect created when ultra-processed foods replace legumes, whole grains, vegetables, fruits and meals based on minimally processed ingredients [55]. Preserving minimally processed food matrices is relevant because food structure can influence nutrient release, fibre accessibility, microbial fermentation and the way fibres and polyphenols interact with the gut environment [146]. A randomised controlled trial comparing different almond forms showed that almond processing influenced gastrointestinal microbiota composition, supporting the idea that the physical structure of a food can affect microbial responses even within the same food category [148].

Fresh fruits, vegetables and fermented foods may also contribute food-associated microorganisms and microbial metabolites, although these effects should be framed carefully. For this reason, fermented foods should be considered part of microbiome-conscious dietary patterns, provided that their use remains compatible with food safety and appropriate processing conditions [28]. Eating foods that contain live microbes does not necessarily mean that those microbes will permanently colonise the gut. Their contribution may instead lie in repeated exposure, transient activity, metabolic inputs and support for microbial diversity [5,28]. A controlled dietary intervention showed that a high-fermented-food diet steadily increased gut microbial diversity and reduced several inflammatory markers, whereas a high-fibre diet mainly increased microbiome-encoded carbohydrate-active enzymes, with immune responses depending partly on baseline microbial diversity [39]. Food safety remains essential. The goal is not to increase microbial exposure indiscriminately, but to better understand which food-system practices can preserve safe and potentially beneficial microbial diversity [25]. This also raises a broader cultural and ecological question: which food practices, plant uses and local knowledge systems help maintain dietary diversity and safe microbial exposure?

Microbiome-conscious food systems should not overlook traditional and local knowledge, especially where it relates to edible biodiversity, fermented foods, medicinal plants, land management and biodiversity stewardship [149]. Recent discussions at the World Health Assembly have framed traditional, complementary and integrative medicine within broader questions of evidence, regulation, equity and planetary health [150]. In this review, the relevance of these approaches lies in their potential links with dietary diversity, microbial exposure and culturally rooted health behaviours, provided they are assessed through evidence-informed, quality-assured and safety-oriented frameworks [39,151,152].

Finally, microbiome-conscious food systems will require better integration between fields that are often studied separately. Soil science, food microbiology, nutrition, ecology and public health need to be connected through longitudinal studies that follow microbial communities and functions across the soil–food–gut pathway [25]. This would help clarify which microbes persist, which are lost, and which microbial functions actually matter for resilience. In this sense, microbial stewardship can be understood as a One Health responsibility: maintaining the ecological conditions that support beneficial microbial communities and their function across soils, foods, ecosystems and hosts [9].

## 7. Conclusions

The gut microbiome is shaped by diet, but diet itself is shaped by broader ecological and food-system conditions [7]. Soils, plants, foods, animals and humans are connected through nutrients, microbial substrates, metabolites, environmental exposures and microbial communities [6]. From this perspective, food is not only defined by its nutritional composition, but also by the ecological conditions that shape it.

At the same time, this continuum should be interpreted with caution [6]. The links between soil microbiomes, food-associated microbes and the human gut are still being clarified, and microbial exposure through food does not necessarily imply stable colonisation or direct health effects [25]. Even so, the available evidence supports a broader view of gut microbiome resilience, one that considers upstream ecological and food-system conditions.

Modern food systems may influence this farm-to-fork pathway through agricultural intensification, processing, dietary simplification, antimicrobial pressure and environmental pressures [24]. A microbiome-conscious One Health approach should therefore move beyond the control of microbial risks alone and also consider how beneficial microbial diversity and function can be protected across ecosystems.

Ultimately, microbial stewardship offers a useful direction for future research and practice [9]. Taken together, evidence from early-life microbiome research, diet–microbiome studies, agricultural microbiome studies, pollutant-exposure models and food additive cohorts suggest that microbiome resilience is shaped across several connected environments, not only within the intestine. The key challenge is not to maximise microbial exposure, but to understand which microbial functions matter, where they are maintained or lost, and how food systems can support them and strengthen resilience without compromising food safety.

## Figures and Tables

**Table 1:** Examples of microbiome-conscious interventions and indicators across the food-system continuum.

Intervention Level	Example Action	Microbiome-Related Indicator	Health/Environment Indicator
Soil/agriculture	Cover crops, compost, reduced tillage	Soil microbial diversity/function	Soil organic carbon, crop resilience
Food processing	Preserve safe live microbial foods	Food microbiome profiling	Food safety and shelf life
Diet	Diverse plant-rich diets, including fibre-rich minimally processed foods, legumes and fermented foods	Skin/gut microbiota diversity, environmental microbiome exposure	Metabolic/inflammatory markers
Environmental exposure	Biodiverse schoolyards, urban green spaces, contact with soil/vegetation	Skin/gut microbiota diversity, environmental microbiome exposure	Immune regulation, allergy/asthma-related indicators
Antimicrobial resistance	Reduce unnecessary antimicrobial use	Resistome monitoring	AMR burden
Animal production	Prudent antimicrobial use, vaccination, improved biosecurity, manure treatment	Animal gut microbiome and resistome; manure resistome	Reduced antimicrobial use, lower AMR burden, animal welfare, safer manure recycling

## Data Availability

No new data were created or analyzed in this study. Data sharing is not applicable to this article.

## Abbreviations

The following abbreviation is used in this manuscript:

AMR	Antimicrobial Resistance
